# Mutation-resolved ABL1 inhibitor resistance prediction under mutation-disjoint and temporal cross-study validation

**DOI:** 10.1371/journal.pone.0353101

**Published:** 2026-10-27

**Authors:** Mohanad A. Deif, Mohamed A. Hafez, Mohammad Khishe, Ahmed Solyman, Ismail A. M. Elhaty

**Affiliations:** 1 Research Institute of Sciences and Engineering, University of Sharjah, Sharjah, United Arab Emirates; 2 Department of Computer Science, College of Information Technology, Misr University for Science and Technology (MUST), Giza, Egypt; 3 Faculty of Engineering, INTI International University, Nilai, Malaysia; 4 Shinawatra University, Bangkok, Pathum Thani, Thailand; 5 Applied Science Research Center, Applied Science Private University, Amman, Jordan; 6 Department of Engineering, School of Science and Engineering, Glasgow Caledonian University, Glasgow, United Kingdom; 7 Istanbul Gelisim University, Istanbul, Turkey; Centre de Recherche en Biologie cellulaire de Montpellier, FRANCE

## Abstract

Computational prediction of ABL1 tyrosine-kinase inhibitor (TKI) resistance is difficult because available mutation–drug datasets are small, imbalanced, and heterogeneous. This study evaluates structure-aware resistance prediction under mutation-disjoint internal validation and temporal cross-study outcome validation. The development benchmark comprised 144 ABL1 mutation–TKI records (19 resistant and 125 susceptible) labelled by a ten-fold affinity-loss criterion. A fixed panel of 11 documented structural and energetic predictors was used to minimize data-adaptive feature selection within the validation framework. A calibrated ensemble combining Elastic-Net logistic regression, radial-basis-function support-vector machines, random forests, and XGBoost was evaluated using mutation-disjoint five-fold grouped out-of-fold prediction repeated ten times. Temporal validation used a later prime-editing resistance map, yielding 84 exact mutation–TKI matches across imatinib, nilotinib, bosutinib, and ponatinib; 61 had binary Sensitive/Resistant outcomes and 23 were retained as Intermediate. The 11-feature stack achieved grouped internal AUROC 0.645 (95% CI 0.429–0.830) and PR-AUC 0.230, with mean ± SD AUROC 0.583 ± 0.101 across repeated grouped splits. On the 61 binary later-study outcomes, AUROC was 0.605 (95% CI 0.431–0.874) and PR-AUC 0.835, modestly above the resistance-prevalence baseline of 0.770. External calibration was poor (Brier score 0.583; calibration slope 0.441). Elastic-Net showed somewhat higher temporal discrimination (AUROC 0.643; PR-AUC 0.877). These results indicate limited transportability across mutation identities and experimental platforms and support use of the framework for hypothesis-generating resistance screening rather than clinically calibrated treatment selection.

## Introduction

The introduction of imatinib established BCR–ABL1 inhibition as a model of molecularly targeted cancer therapy and markedly improved outcomes in chronic myeloid leukemia (CML) [1,2]. Successive ATP-site inhibitors and the allosteric inhibitor asciminib expanded treatment options, including activity against several mutations that compromise earlier therapies [3,4]. Nevertheless, BCR–ABL1 kinase-domain mutations remain an important cause of treatment failure, and international recommendations retain mutation testing as a component of therapeutic decision making in appropriate clinical settings [5–7].

Resistance is mutation–drug specific rather than an invariant property of an amino-acid substitution. A variant may alter steric complementarity, hydrogen-bond networks, solvent exposure, conformational equilibria, or binding free energy differently across TKIs [8,9]. Experimental characterization of every possible mutation–inhibitor combination is costly, motivating computational methods that can prioritize variants for confirmatory testing. The central challenge is not only predictive discrimination within a historical dataset, but also transportability across mutation identities, time, and experimental platforms.

## Literature review

### ABL1 kinase-domain resistance and treatment context

Clinical and mechanistic studies established that kinase-domain substitutions can reduce inhibitor binding directly, stabilize alternative kinase conformations, or generate cross-resistance across sequential therapies [3,5,7]. The gatekeeper T315I substitution is a prominent example, but resistance spectra extend beyond a single locus and vary among imatinib, second-generation inhibitors, ponatinib, and asciminib [3,4]. Contemporary CML guidance therefore interprets mutational status together with treatment history, disease phase, comorbidities, and available therapies rather than treating a mutation as an isolated deterministic marker [6]. This context sets an appropriate role for computational prediction: prioritization and mechanistic hypothesis generation, not replacement of clinical or experimental evidence.

### Physics-based and structure-guided prediction

Physics-based calculations offer a mechanistically grounded estimate of mutation-induced affinity change. Using a benchmark of 144 clinically observed ABL mutation–TKI pairs, Hauser et al. reported that alchemical free-energy perturbation could classify resistance with approximately 88% accuracy and estimate mutation-induced binding free-energy changes with an RMSE of approximately 1.1 kcal mol^–1^ [8]. Related structure-based workflows use docking, force-field energies, solvent accessibility, residue-environment descriptors, and molecular dynamics to characterize binding determinants [10–12]. For example, Dhanasekaran et al. combined docking and molecular dynamics to investigate flavonoid–protein interactions in an oncology setting, illustrating how complementary simulations can probe the stability and mechanistic plausibility of predicted complexes [13]. Such workflows are informative but computationally intensive and sensitive to structural preparation and sampling choices.

Structure-guided machine learning provides a lower-cost alternative once descriptors are available. mCSM-lig formalized graph-based structural signatures for mutation-induced changes in protein–small-molecule affinity [14]; SUSPECT-ABL and SPLDExtraTrees subsequently combined physicochemical, structural, interaction, and energetic information for kinase-inhibitor resistance prediction [9,15]. More broadly, machine learning has become central to ligand screening and drug-target prediction, although benchmark gains do not automatically imply prospective utility [16,17]. Consensus modelling can integrate complementary virtual-screening signals; the holistic framework of Moshawih et al., for example, demonstrated a machine-learning strategy for combining diverse screening evidence [18]. These studies motivate ensemble learning, while also underscoring the need to test whether added complexity improves performance under genuine distribution shift.

### Validation, calibration, and transportability

Small molecular datasets are especially vulnerable to optimistic evaluation when closely related compounds, mutation identities, or replicated assay contexts are divided between training and test partitions. Ligand-based benchmarks can reward memorization rather than generalization, and model selection conducted outside nested resampling can bias error estimates [19,20]. Time-split validation more closely approximates prospective deployment, while repeated cross-validation is necessary to expose the large uncertainty caused by small samples [21,22]. For imbalanced outcomes, PR-AUC should be interpreted relative to prevalence rather than in isolation [23]. Furthermore, good ranking does not guarantee reliable probabilities; calibration must be evaluated explicitly whenever outputs could be interpreted as risk estimates [24].

Published work on the same 144-record ABL1 benchmark has evaluated leakage-controlled stacked learning and identified a compact set of informative structural predictors [25]. Complementary public-bioactivity work has harmonized BindingDB and ChEMBL mutation–drug records and shown that performance declines under source-aware database shift [26]. A later prime-editing study produced a substantially broader experimental map of ABL1 variant responses to five TKIs, providing an independent temporal outcome source for assessing whether historical predictions retain value across assay platforms [27].

## Research gap and study contributions

Existing ABL1 resistance studies establish clinical relevance and demonstrate the feasibility of physics-based and machine-learning prediction, but four connected gaps remain. First, record-level splitting can place different drug records from the same mutation in both training and evaluation data. Second, feature discovery and performance estimation are often conducted on the same small benchmark, obscuring the distinction between panel selection and model validation. Third, later-study experimental outcomes are rarely used to quantify temporal cross-study transport. Fourth, discrimination is commonly emphasized without parallel assessment of calibration, prevalence-aware precision–recall performance, group-level uncertainty, and decision-threshold transfer. To our knowledge, these elements have not previously been evaluated together for this historical ABL1 structural benchmark and the later prime-editing atlas.

This study makes four specific contributions:

1. it evaluates a fixed and fully documented 11-descriptor structural panel, preserving a clear boundary between historical feature selection and later-study assessment;
2. it implements repeated, nested, mutation-disjoint grouped OOF stacking in which preprocessing, meta-learning, and probability calibration are restricted to training partitions;
3. it performs exact mutation–TKI mapping to a temporally subsequent prime-editing dataset, while explicitly distinguishing temporal outcome transport from unseen-variant input generalization; and
4. it jointly reports AUROC, prevalence-referenced PR-AUC, probability calibration, development-only threshold transfer, repeated-split stability, and mutation-cluster bootstrap uncertainty, with record-level outputs and reproducible code.

The primary research question is therefore whether structure-aware predictions learned from the historical benchmark retain discrimination and calibration when mutation identities are separated during internal validation and outcomes are assessed against a later experimental platform.

## Materials

### Study design and data sources

The study combined a historical structure-aware ABL1–TKI benchmark for model development with a temporally subsequent prime-editing dataset for cross-study outcome evaluation. Historical data were used for model fitting and out-of-fold (OOF) prediction, whereas Jung et al. classifications were used only as later experimental outcomes. No Jung classification, resistance score, or prevalence information entered predictor selection, imputation, scaling, model fitting, calibration, or decision-threshold selection. The mapped Jung records correspond to mutation–drug identities present in the historical feature matrix and therefore evaluate temporal outcome transport rather than completely unseen structural inputs (Fig 1).

**Fig 1:**
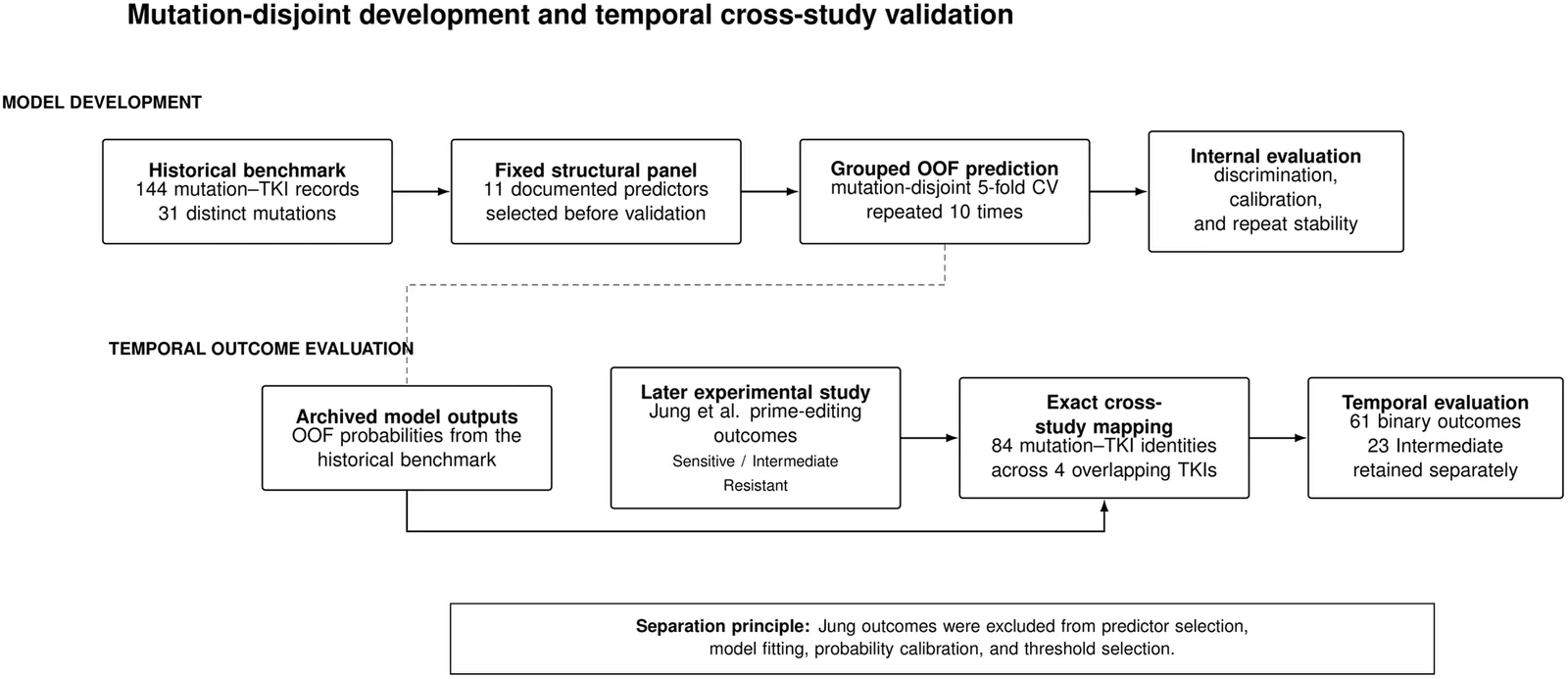
Study design for mutation-disjoint and temporal cross-study validation. The 144-record historical benchmark was represented by a fixed 11-predictor structural panel and evaluated by mutation-disjoint grouped OOF prediction. Jung outcomes were mapped by exact mutation–TKI identity. The primary temporal analysis used 61 binary Sensitive/Resistant outcomes, while 23 Intermediate outcomes were retained separately.

### Historical ABL1–TKI benchmark and outcome definition

The development benchmark contained 144 ABL1 mutation–TKI records representing 31 distinct mutation identities across eight inhibitors. The continuous response variable was the logarithm of the fold change in inhibitor affinity relative to wild type. Following the benchmark definition, resistance was defined as a ten-fold or greater loss of affinity, corresponding to $\log_{10}(fold\;change)>1$ and approximately ΔΔG≥1.36 kcal mol^–1^ [8]. This definition yielded 19 resistant and 125 susceptible records. The binary label was used only as the supervised endpoint and was not included among model predictors. No synthetic oversampling or feature-space interpolation was used for model validation.

### Structural descriptor provenance

The TKI feature matrix originates from the public SPLDExtraTrees benchmark [15]. The full matrix contains ligand, mutation, local-environment, protein–ligand interaction, AutoDock-Vina, solvent-accessible surface area (SASA), and Rosetta energy descriptors. In the source workflow, ligand physicochemical properties were derived from RDKit representations; mutation-level physicochemical descriptors and FoldX-derived stability terms represented residue changes; local environments encoded geometry and residue composition around the mutated position; PLIP summarized protein–ligand contacts; Vina-derived scoring terms and SASA descriptors represented protein–ligand energetic and exposure properties; and Rosetta REF15 terms described all-atom energetic contributions [10–12,15,28]. These descriptor families are consistent with the broader use of structure-derived signatures to model mutation effects on protein–small-molecule affinity [14]. PDB identifiers and ligand identifiers are retained at record level in S1 Data, and the source repository provides the archived feature matrix and analysis notebooks used to generate the published benchmark. The present study used these archived values without recomputing structure preparation, docking, Rosetta, FoldX, SASA, or conformational ensembles. Consequently, uncertainty from upstream protonation choices, binding-site preparation, docking grids, force-field settings, and conformational sampling cannot be estimated independently here and is treated as a limitation rather than absorbed into model uncertainty.

### Fixed 11-predictor structural panel

The primary analysis used a fixed set of 11 structural predictors reported as FDR-significant in published work on the same benchmark [25]. The variables were specified before temporal validation and are listed in Table 1. Because this feature panel was originally identified from the historical benchmark, the internal OOF analysis estimates the robustness of a fixed panel rather than a fully nested estimate of feature discovery. Jung classifications and resistance scores were not used to define or modify the predictor panel.

**Table 1:** Fixed 11-predictor structural panel used for model development and validation.

Family	Predictor	Reported resistant-record pattern
Local environment	ENV.LigRDF_all_2.0_3.0	Higher
Local environment	ENV.LigAng_Q4	Higher
Local environment	ENV.LigDist_Q0	Lower
Protein–ligand interaction	PLIP.HBOND	Distributional shift
AutoDock Vina	VINA.F1	Lower
AutoDock Vina	VINA.F51	Distributional shift
AutoDock Vina	VINA.F52	Distributional shift
AutoDock Vina	VINA.F53	Distributional shift
SASA	SASA.AR	Lower
SASA	SASA.PL	Lower
Rosetta	yhh_planarity	Higher

### Temporal cross-study dataset and exact mapping

Jung et al. used prime editing to construct a near-comprehensive ABL1 single-amino-acid-variant resistance map and evaluated five TKIs: imatinib, nilotinib, bosutinib, ponatinib, and asciminib [27]. Four drugs overlapped the historical structural benchmark. Exact matching by amino-acid substitution and TKI identified 84 historical feature vectors spanning 21 mutation identities and the four overlapping drugs. The later-study classifications comprised 47 Resistant, 14 Sensitive, and 23 Intermediate records.

The prespecified primary temporal analysis compared Resistant with Sensitive outcomes only (*n* = 61). Intermediate records were retained as a separate descriptive class and excluded from the binary endpoint. Historical affinity-based labels and Jung experimental classifications were stored in separate variables because they represent different outcome systems. The 84 matched records were evaluated with historical OOF probabilities, not probabilities from a model fitted to all 144 historical records. This analysis therefore evaluates temporal cross-study outcome transport for exact mutation–TKI matches; it does not establish generalization to entirely unseen variant feature vectors. That stronger test would require computation of the same structural descriptors for Jung-only variants.

## Methodology

### Ethics statement

Not applicable. This study used previously published computational and experimental datasets and did not involve new human participants, identifiable human data, or animals.

### Preprocessing and model specification

All transformations were estimated within the relevant training partition. Missing numerical values, when present, were replaced by the training-fold median. Elastic-Net logistic regression and the radial-basis-function support-vector machine (RBF-SVM) were standardized using training-fold means and standard deviations; tree-based models were fitted to the median-imputed values without z-score scaling. Class imbalance was handled through model-specific weighting where supported rather than through synthetic resampling.

The ensemble contained four base learners: Elastic-Net logistic regression, RBF-SVM, random forest, and XGBoost. Elastic-Net logistic regression used *C* = 0.7, *l*_1_ ratio = 0.5, balanced class weights, and the SAGA optimizer; the RBF-SVM used *C* = 1.0, γ set to the scikit-learn “scale” rule, and balanced class weights; the random forest used 120 trees, a minimum leaf size of 3, and balanced-subsample weighting; and XGBoost used 70 trees, maximum depth 2, learning rate 0.04, subsample fraction 0.85, column-subsample fraction 0.85, *L*_2_ regularization 2.0, and *L*_1_ regularization 0.1. Base-model probabilities were combined by an *L*_1_-regularized logistic meta-learner (*C* = 0.8). These settings were specified independently of the Jung outcomes. Individual base learners were evaluated on the same outer folds as benchmark models.

### Mutation-disjoint grouped OOF prediction

The primary internal validation used five-fold stratified grouped cross-validation repeated ten times. Mutation identity was the grouping variable. Consequently, all records associated with a given mutation were allocated to the same outer test fold in a given repeat, and no copy of that mutation appeared in the corresponding outer training partition. The split therefore tests transfer to held-out mutation identities rather than merely to held-out mutation–drug rows. This grouping strategy addresses identity-based memorization, while repetition exposes split sensitivity in the small benchmark [19,22].

Within each outer training partition, grouped inner folds were used to create base-learner OOF predictions for meta-learner fitting. The base learners were subsequently refitted to the complete outer-training partition and used to generate probabilities for the outer-test records. The fitted meta-learner combined those outer-test base probabilities. A Platt-style logistic calibrator was estimated from training-partition OOF ensemble scores only, then applied to the outer-test ensemble score. Each historical record was therefore predicted only while its mutation group was held out. The ten held-out probabilities obtained across the repeated grouped splits were averaged to produce one final OOF probability per record.

The ensemble using the fixed structural panel is referred to as the *11-feature stack*.

### Mathematical formulation and pseudocode

Let the historical dataset be


$$\begin{array}{c} 𝒟=\{({𝐱}_{i},y_{i},g_{i}{\}}_{i=1}^{n}, \end{array}$$ (1)

where ${𝐱}_{i}\in {\mathbb{R}}^{11}$ is the fixed structural descriptor vector, $y_{i}\in \{0,1\}$ is the affinity-based resistance label, and $g_{i}$ is the mutation identity. For repeat r∈{1,...,R} and outer fold k∈{1,...,K}, the grouped partition ${𝒯}_{rk}\cup {𝒱}_{rk}=𝒟$ satisfies


$$\begin{array}{c} \{g_{i}:i\in {𝒯}_{rk}\}\cap \{g_{i}:i\in {𝒱}_{rk}\}=\emptyset ,\;R=10,\;K=5. \end{array}$$ (2)

For each held-out record $i\in {𝒱}_{rk}$, the four base learners produce probabilities $p_{im}^{\left(r\right)}$, m=1,...,4. The training-only logistic meta-learner produces


$$\begin{array}{c} s_{i}^{\left(r\right)}=\sigma \;\left(\beta_{0}^{\left(rk\right)}+\sum_{m=1}^{4}\beta_{m}^{\left(rk\right)}p_{im}^{\left(r\right)}\right),\;\sigma (u)=\frac{1}{1+e^{-u}}. \end{array}$$ (3)

A training-only Platt-style calibrator maps this score to


$$\begin{array}{c} {\tilde{p}}_{i}^{\left(r\right)}=\sigma \;\left(a_{rk}s_{i}^{\left(r\right)}+b_{rk}\right), \end{array}$$ (4)

and the final historical OOF probability is the average across repeats,


$$\begin{array}{c} {\overset{―}{p}}_{i}=\frac{1}{R}\sum_{r=1}^{R}{\tilde{p}}_{i}^{\left(r\right)}. \end{array}$$ (5)

The same archived ${\overset{―}{p}}_{i}$ values were then linked to later-study outcomes by exact mutation–TKI identity. No later-study label was used to estimate β, *a*, *b*, or an operating threshold.

**Algorithm 1. Mutation-disjoint grouped OOF stacking and temporal evaluation.**

**Input:** Historical records 𝒟; fixed descriptor panel; later-study table 𝒥; base learners $f_{1},...,f_{4}$; *R* = 10 repeats; *K* = 5 folds.

**Output:** Aggregated OOF probabilities $\overset{―}{p}$, development thresholds, and temporal validation statistics.

1. For each repeat *r*, construct stratified folds of mutation groups so that no $g_{i}$ occurs in both ${𝒯}_{rk}$ and ${𝒱}_{rk}$.

2. For each outer fold *k*, estimate imputation and scaling on ${𝒯}_{rk}$ only.

3. Generate grouped inner-OOF base probabilities within ${𝒯}_{rk}$; fit the logistic meta-learner and calibrator using only these training-partition predictions.

4. Refit each $f_{m}$ on all of ${𝒯}_{rk}$, predict ${𝒱}_{rk}$, apply the fitted meta-learner and calibrator, and store ${\tilde{p}}_{i}^{\left(r\right)}$.

5. Average repeated held-out probabilities: ${\overset{―}{p}}_{i}\leftarrow R^{-1}\sum_{r}{\tilde{p}}_{i}^{\left(r\right)}$.

6. Select $\tau_{q}=\text{arg}\max_{\tau :\;Sensitivity(\tau )\geq q}MCC(\tau )$ from historical OOF predictions only, for q∈{0.70,0.90}.

7. Exact-match $\overset{―}{p}$ to 𝒥 by mutation–TKI identity; retain Sensitive/Resistant records for the primary binary endpoint and Intermediate records for descriptive analysis.

8. Estimate discrimination, calibration, and threshold metrics; resample mutation groups for bootstrap uncertainty and paired model contrasts.

### Development-only threshold selection

Threshold selection was performed exclusively on the aggregated historical OOF predictions. Two sensitivity-constrained operating rules were defined a priori, requiring sensitivity ≥0.90 and ≥0.70, respectively. For each rule, the decision threshold τ was chosen to maximize the Matthews correlation coefficient among thresholds satisfying the relevant sensitivity floor. Jung outcomes were not used to choose or modify τ. AUROC and PR-AUC were the primary temporal performance measures because they do not depend on a single operating threshold.

### Performance measures and calibration

Discrimination was summarized by area under the receiver-operating-characteristic curve (AUROC) and area under the precision–recall curve (PR-AUC). For the temporal binary set, the resistant prevalence was 47/61 = 0.770 and was reported as the no-skill reference for PR-AUC interpretation, as recommended for imbalanced classification [23]. Threshold-dependent measures included accuracy, balanced accuracy, precision, sensitivity, specificity, negative predictive value, F1 score, and MCC. Probabilistic performance was assessed by Brier score and log loss. Calibration was further evaluated using calibration bins and by fitting a logistic recalibration model to estimate calibration intercept and calibration slope; values near 0 and 1, respectively, indicate ideal calibration [24].

### Uncertainty analysis and repeated-split stability

Confidence intervals were calculated with 1,000 percentile bootstrap resamples at the mutation-cluster level rather than at the individual row level. Resampling mutation identities preserves dependence among multiple TKI records associated with the same mutation. Bootstrap intervals were reported for discrimination and threshold metrics when estimable. In addition, the AUROC of the 11-feature stack was calculated separately for each of the ten grouped repeats to quantify sensitivity to group allocation. This repeated-split distribution was treated as a stability diagnostic rather than as an additional source of independent samples.

Paired mutation-cluster bootstrap comparisons with 2,000 resamples were used to quantify AUROC differences between selected models. Two-sided bootstrap *p* values were calculated as twice the smaller empirical tail probability of the paired AUROC difference. These comparisons were treated as descriptive model contrasts because the number of independent mutation groups was limited.

### Physics-based context and scope of comparison

The historical benchmark was originally assembled for alchemical free-energy calculations. Hauser et al. reported approximately 88% resistance-classification accuracy and an RMSE of approximately 1.1 kcal mol^–1^ for mutation-induced binding free-energy changes [8]. Those published FEP results were generated under a different computational and validation workflow and are therefore used here as a physics-based contextual benchmark, not as a paired head-to-head statistical comparison. The current study does not claim that the structure-aware ensemble replaces FEP or molecular dynamics. Rather, the ML analysis tests whether lower-cost structural summaries transport under mutation-disjoint and later-study outcome validation.

### Software and reproducibility

Analyses were conducted in Python 3.13.5 using NumPy 2.3.5, pandas 2.2.3, SciPy 1.17.0, scikit-learn 1.8.0, XGBoost 3.1.3, and Matplotlib 3.10.8. Random seeds were fixed before analysis. Record-level outputs are provided as Supporting Information, including the 144 historical OOF predictions, 84 Jung mappings, the 61-record binary validation subset, model-level metrics, threshold results, calibration bins, and feature-panel documentation. S6 Code recalculates reported discrimination, probability-loss, calibration, and paired-bootstrap summaries from the archived record-level OOF predictions and supplies a reference implementation of the stated grouped OOF training pipeline. Figures were generated from archived result tables without manual alteration of numerical values.

## Results

### Mutation-disjoint validation substantially reduced apparent internal performance

As summarized in Fig 2, under the mutation-disjoint grouped OOF design, the 11-feature stack achieved AUROC 0.645 (95% CI 0.429–0.830), PR-AUC 0.230 (95% CI 0.092–0.523), Brier score 0.117 (95% CI 0.073–0.166), and log loss 0.389 (95% CI 0.271–0.518). Across the ten repeated grouped splits, the mean ± SD stack AUROC was 0.583 ± 0.101, ranging from 0.427 to 0.773 (Fig 3). This variation demonstrates that the small resistant class materially affects performance estimates even when leakage controls are strengthened.

**Fig 2:**
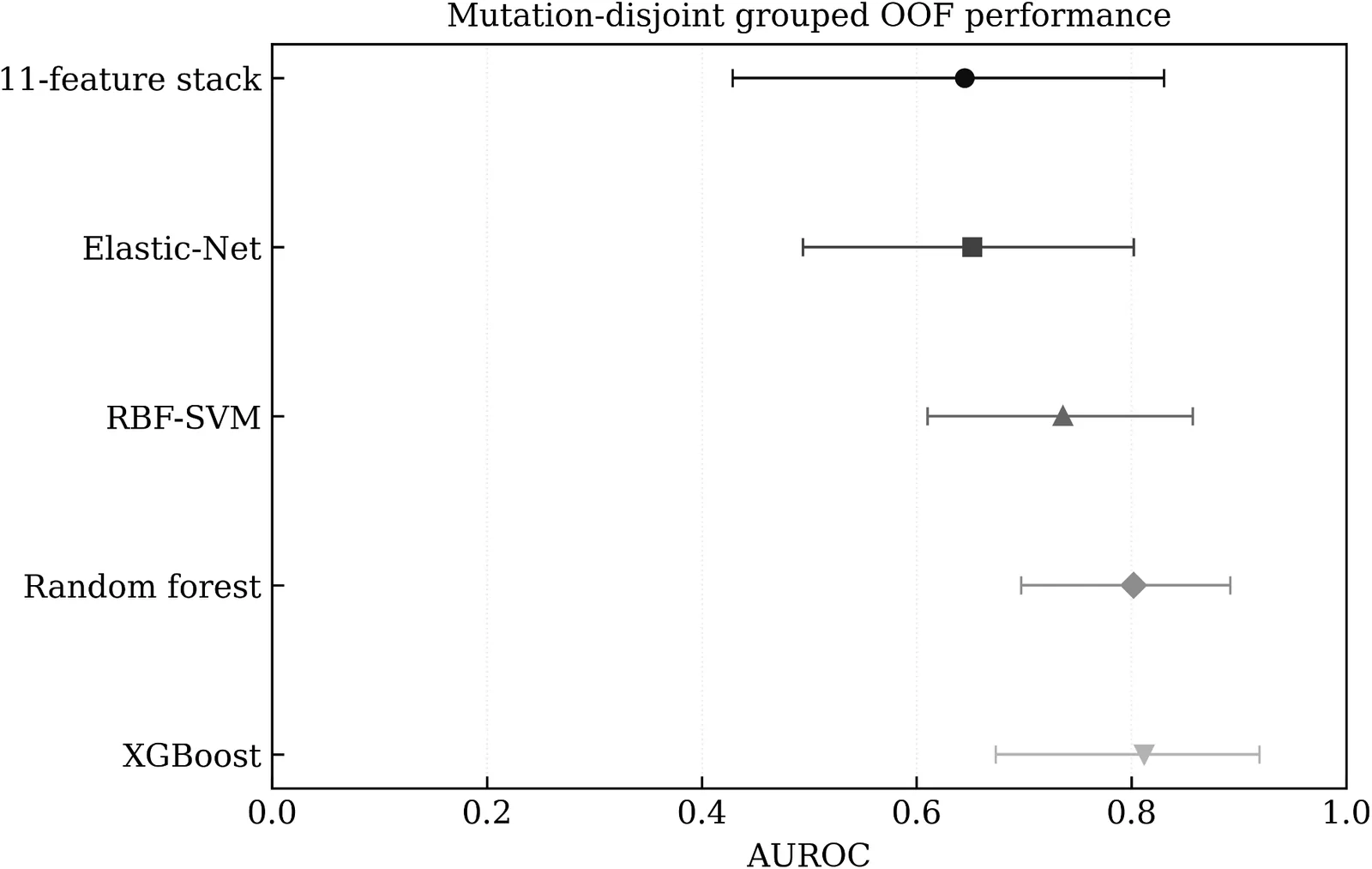
Mutation-disjoint grouped OOF discrimination. Point estimates and mutation-cluster bootstrap 95% confidence intervals for AUROC across the 11-feature stack and individual benchmark learners.

**Fig 3:**
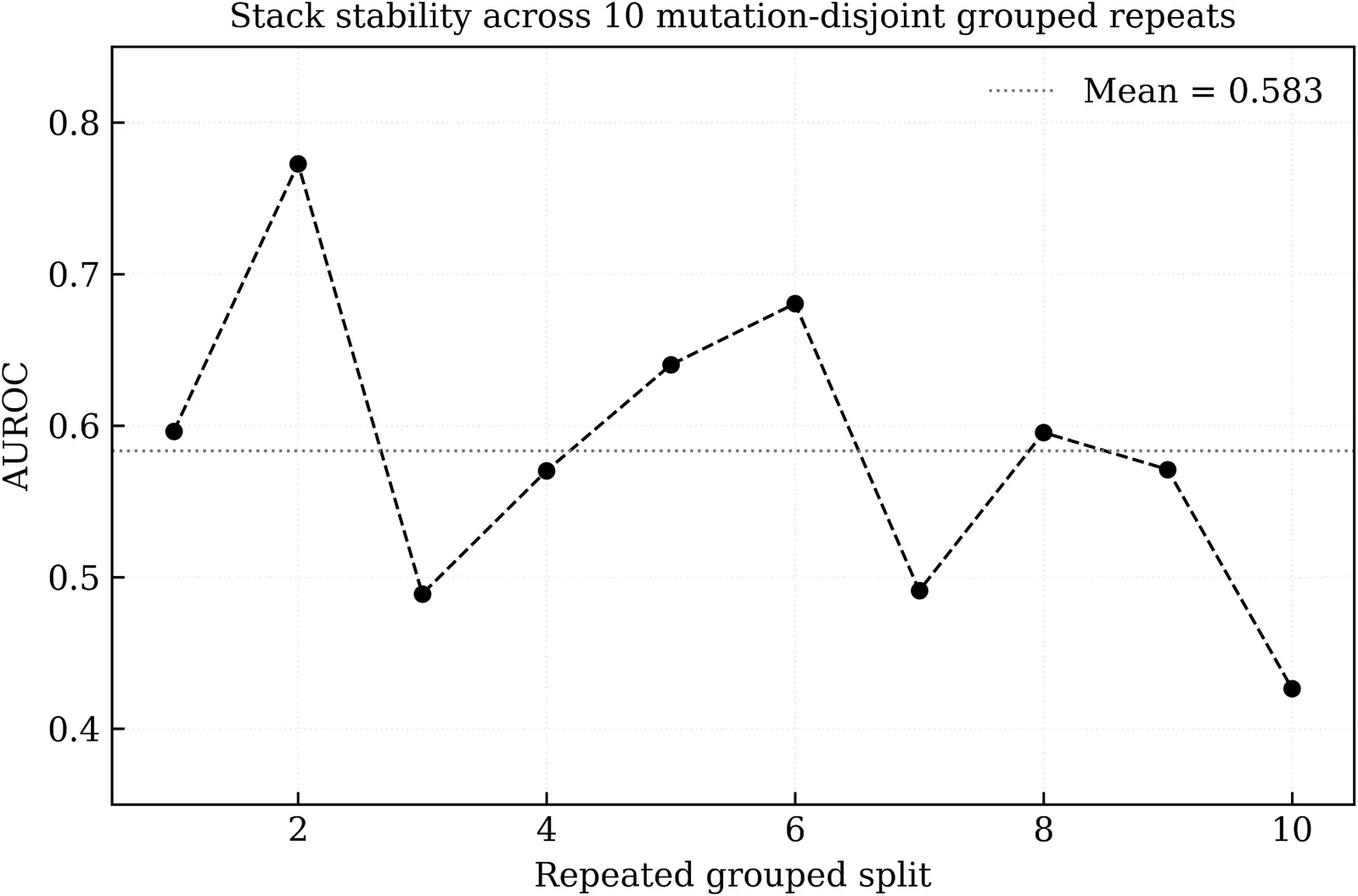
Stability of the 11-feature stack across repeated grouped splits. AUROC is shown for each of the ten mutation-disjoint grouped cross-validation repeats. The horizontal dotted line denotes the mean AUROC of 0.583.

Individual models showed different internal behavior. XGBoost and random forest produced higher grouped OOF AUROCs (0.812 and 0.802, respectively) than the 11-feature stack, whereas their calibration losses did not uniformly improve. In a paired mutation-cluster bootstrap comparison, the XGBoost–stack AUROC difference was 0.167 (95% CI 0.062–0.270; two-sided bootstrap *p* = 0.001). This reinforces the importance of reporting both discrimination and probability quality rather than selecting a model from AUROC alone.

### Internal probability calibration was comparatively favorable

The internal mutation-disjoint OOF calibration curve is shown in Fig 4. Across five quantile bins containing 28–29 records each, predicted probabilities tracked the observed resistant fractions more closely than in temporal validation, with an expected calibration error of 0.046. This estimate nevertheless reflects the historical development distribution and should not be interpreted as evidence of calibration transport to a later experimental platform.

**Fig 4:**
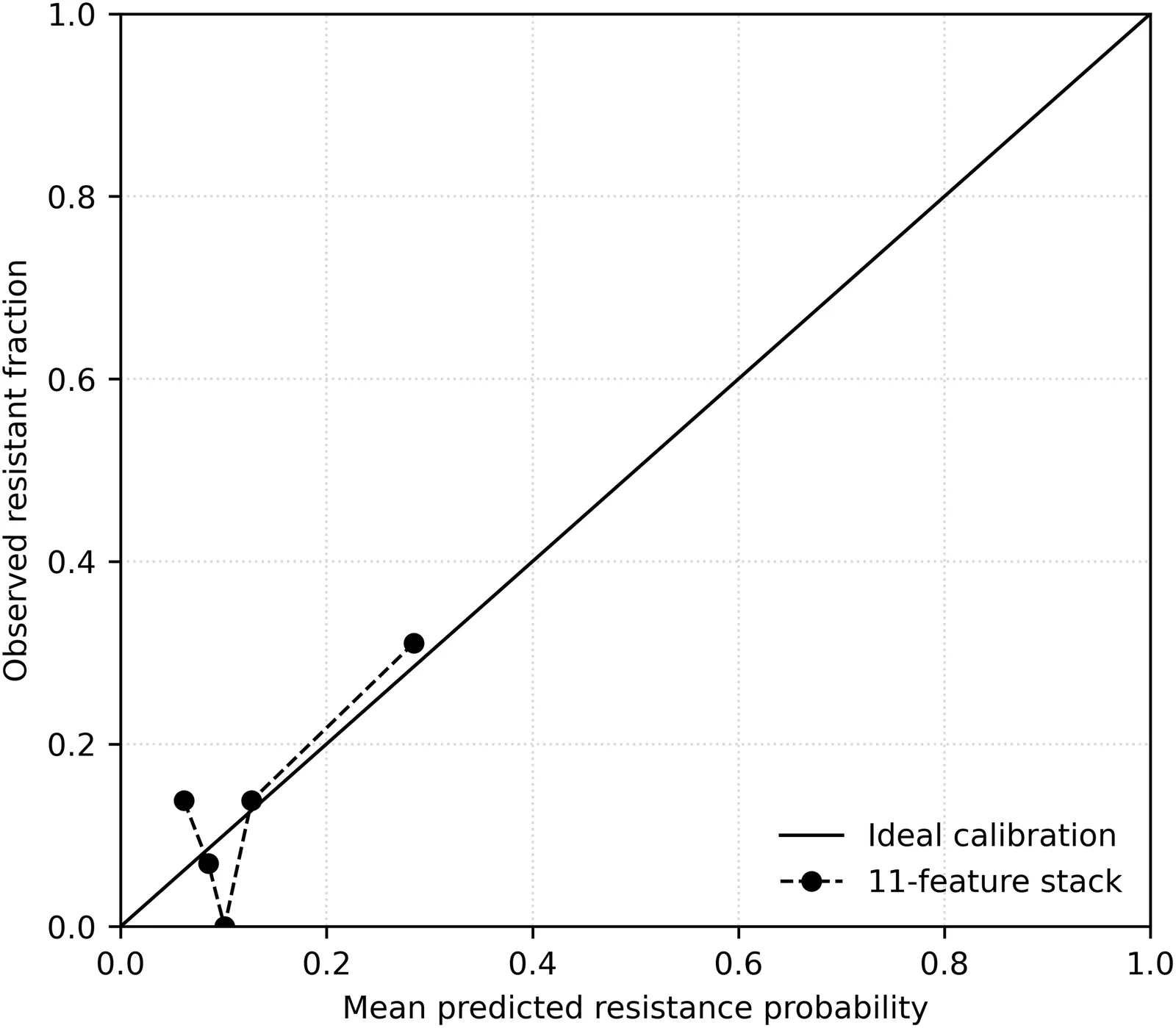
Internal mutation-disjoint OOF calibration of the 11-feature stack. Points show the mean predicted resistance probability and observed resistant fraction within five quantile bins; each bin contained 28–29 records. The solid diagonal denotes ideal calibration.

### Feature-panel stability depended on regularization settings

A secondary Elastic-Net bootstrap sensitivity analysis was conducted within the fixed 11-predictor panel. At the reference setting (*C* = 1.0, *l*_1_ ratio = 0.5), 9 of 11 predictors were selected in at least 80% of 100 stratified bootstrap fits. Across 12 combinations of *C* and *l*_1_ ratio, the number reaching this stability threshold ranged from 3 to 11. The result supports the use of the published panel as a fixed, documented input set while showing that a data-adaptive stability-selected subset would depend materially on the regularization specification (S5 Methods).

### Temporal validation showed modest discrimination with wide uncertainty

Figs 5 and 6 summarize temporal discrimination. Among the 84 exact later-study matches, 61 had binary Jung classifications (47 Resistant and 14 Sensitive). On these records, the 11-feature stack achieved AUROC 0.605 (95% CI 0.431–0.874) and PR-AUC 0.835 (95% CI 0.646–0.988). Because 77.0% of the binary temporal set was resistant, the prevalence baseline for PR-AUC was already 0.770; the absolute gain in PR-AUC was therefore modest.

**Fig 5:**
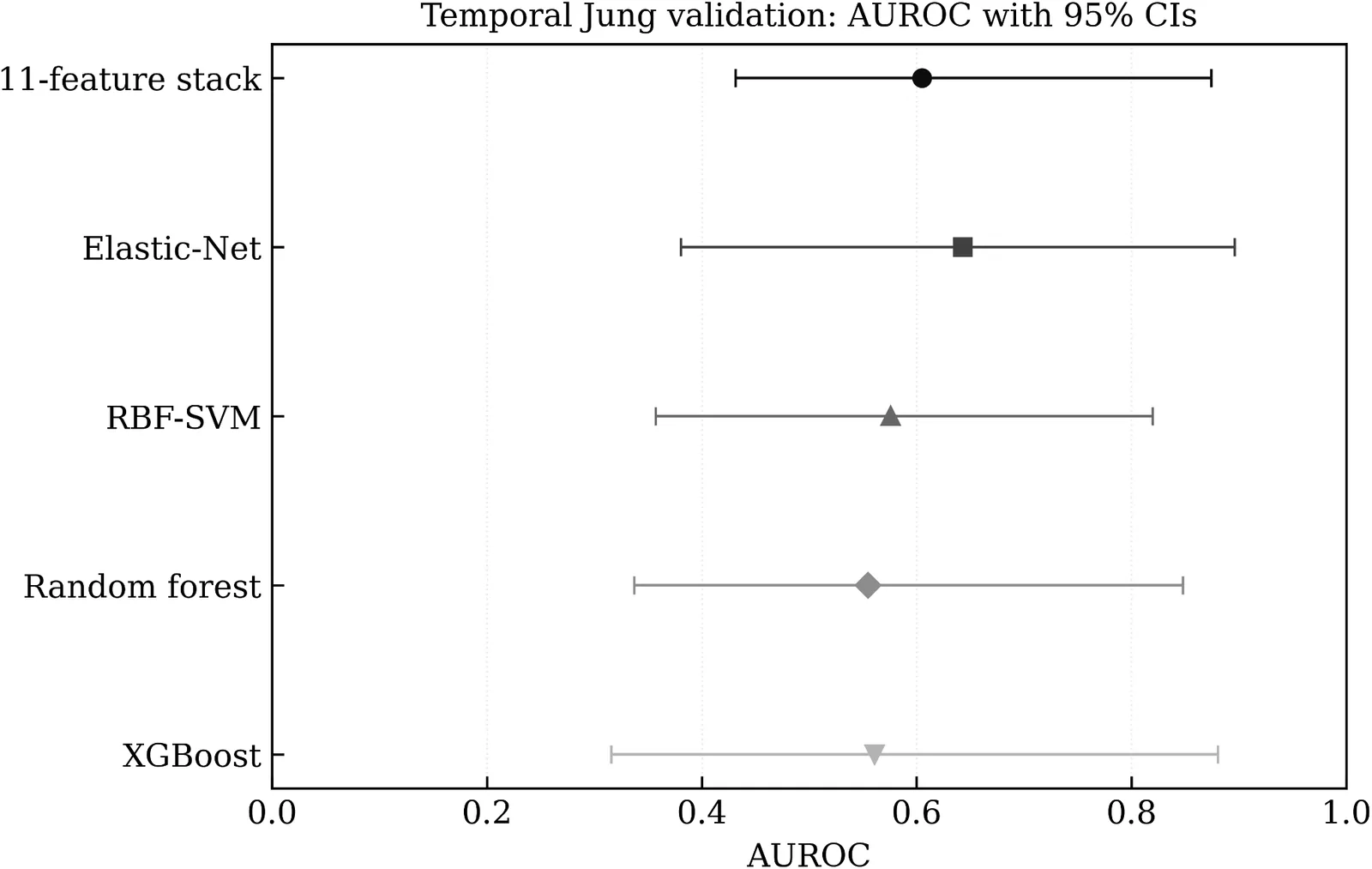
Temporal Jung validation AUROC. Point estimates and mutation-cluster bootstrap 95% confidence intervals across the five models. The wide intervals reflect the limited number of independent mutation groups in the matched external set.

**Fig 6:**
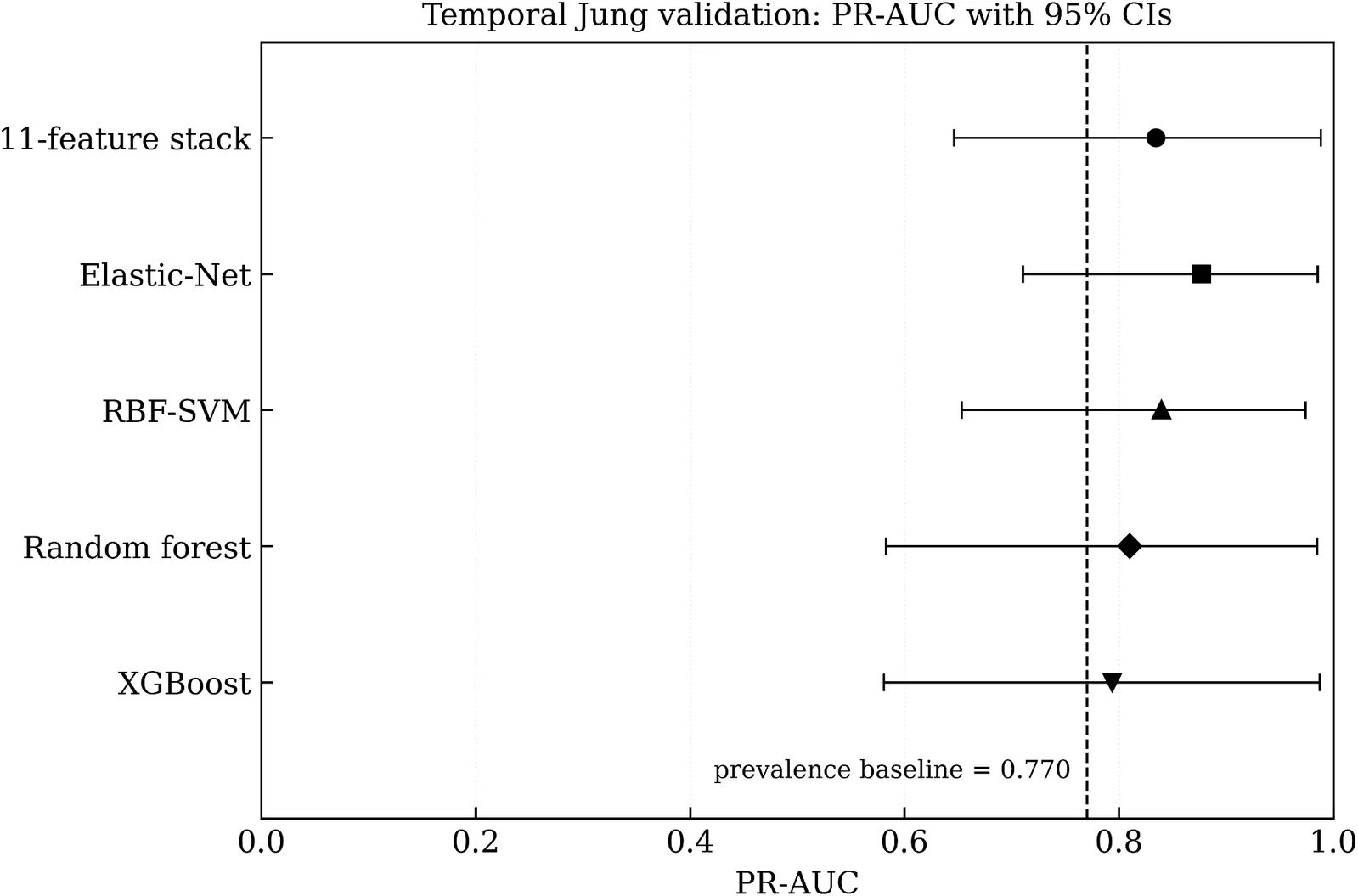
Temporal Jung validation PR-AUC. Point estimates and 95% confidence intervals. The dashed reference marks the resistant prevalence of the binary validation set (47/61 = 0.770), which is the relevant no-skill PR-AUC baseline.

A simpler Elastic-Net model transported somewhat better, with temporal AUROC 0.643 and PR-AUC 0.877. The paired AUROC difference relative to the stack was 0.038 (95% CI −0.233 to 0.262; two-sided bootstrap *p* = 0.793), providing no evidence of a reliable difference between the two models. The other flexible learners showed AUROCs between approximately 0.555 and 0.576. Table 2 summarizes historical grouped OOF and Jung temporal-validation performance for all five models side by side. Thus, complexity did not translate into superior later-study discrimination. This mirrors the broader observation from our separate public-bioactivity study that simpler regularized models can be more robust under distribution shift [26].

**Table 2:** Internal mutation-disjoint and temporal Jung validation performance. Values are point estimates. Confidence intervals for the primary stack are reported in the text and supporting files.

Model	Historical grouped OOF		Jung temporal validation		
	AUROC	PR-AUC	AUROC	PR-AUC	Brier
11-feature stack	0.645	0.230	0.605	0.835	0.583
Elastic-Net	0.652	0.198	0.643	0.877	0.351
RBF-SVM	0.736	0.263	0.576	0.840	0.594
Random forest	0.802	0.306	0.555	0.810	0.521
XGBoost	0.812	0.356	0.561	0.794	0.467

### Probability calibration did not transfer across experimental platforms

The temporal calibration curve is shown in Fig 7. External calibration was substantially worse than the internal calibration shown in Fig 4. For the 11-feature stack, the temporal Brier score was 0.583 (95% CI 0.441–0.712) and log loss was 1.682 (95% CI 1.276–2.061). The calibration intercept was 2.126 and the calibration slope was 0.441, indicating strong distributional shift and miscalibration of historical probabilities relative to the later experimental outcome distribution. Mutation-cluster bootstrap intervals for the calibration intercept (−0.296 to 13.040) and slope (−0.504 to 4.871) were wide, as expected from only 21 mutation groups. In the largest calibration bin, 55 of the 61 binary records had a mean predicted resistance probability of only 0.107, whereas the observed resistant fraction was 0.764.

**Fig 7:**
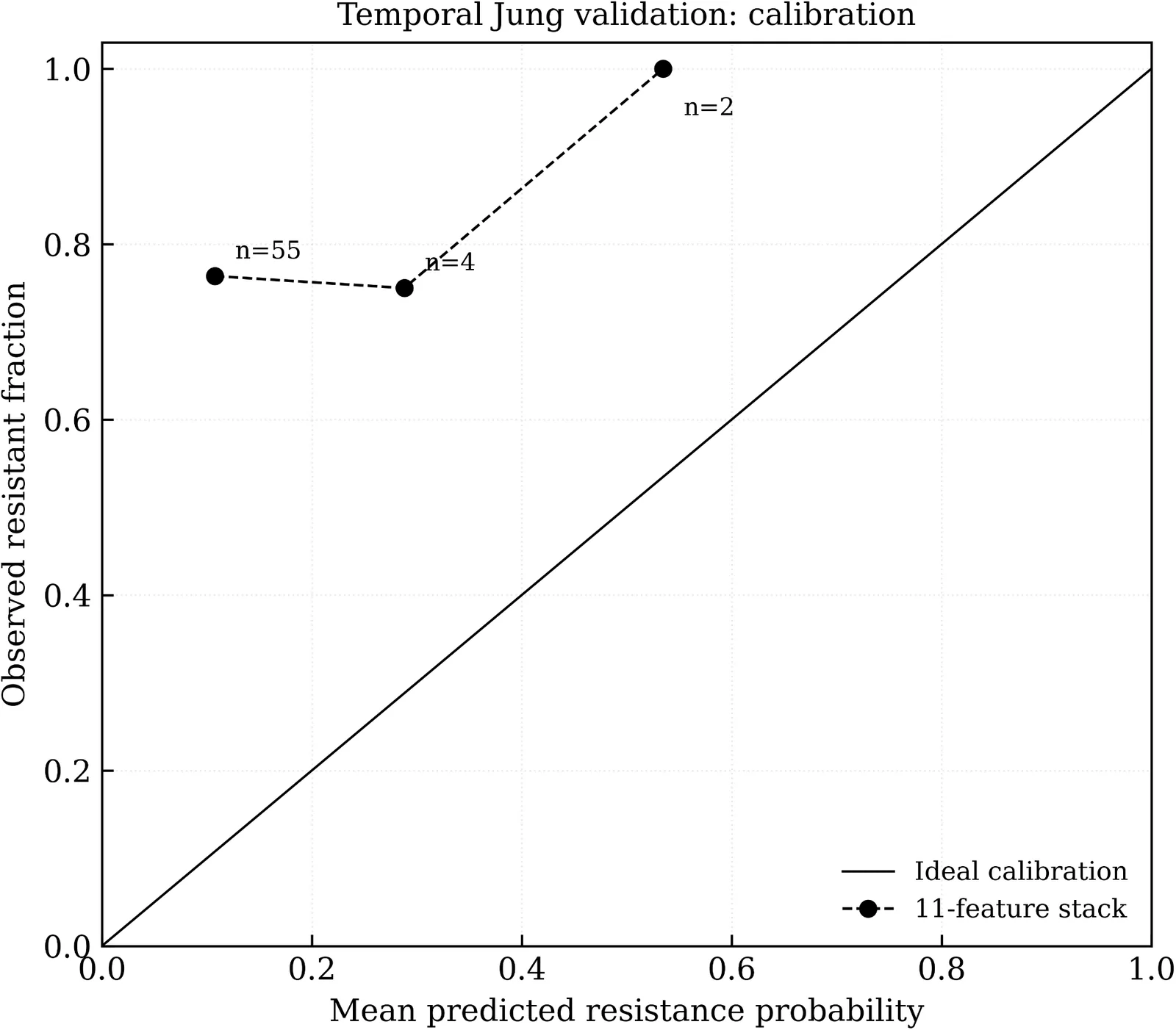
Calibration under temporal cross-study validation. The dashed black model curve is compared with the ideal diagonal. Labels show the number of observations in each populated calibration bin.

These results argue against interpreting the historical model probabilities as clinically calibrated risks. They also illustrate why a later-study calibration assessment is more informative than reporting a low Brier score from within the original development distribution alone.

### Threshold transfer exposed a sensitivity–specificity trade-off

The development-only threshold satisfying sensitivity ≥0.70 while maximizing MCC was τ=0.09. On the temporal binary set, this yielded sensitivity 0.787, specificity 0.500, balanced accuracy 0.644, F1 0.813, and MCC 0.269 (95% CI 0.033–0.581). In contrast, the high-sensitivity development constraint of sensitivity ≥0.90 selected τ=0.04 and achieved temporal sensitivity 0.957 but specificity 0.000. Both operating rules are compared in Table 3. The latter operating rule is therefore not useful as an external decision threshold.

**Table 3:** Temporal performance of thresholds selected only from historical development OOF predictions.

Development rule	Threshold	Sensitivity	Specificity	Balanced accuracy	F1	MCC
Sensitivity ≥0.70	0.09	0.787	0.500	0.644	0.813	0.269
Sensitivity ≥0.90	0.04	0.957	0.000	0.479	0.849	−0.101

### Intermediate outcomes support a distribution-shift interpretation

As shown in Fig 8, the 23 Intermediate Jung classifications were not forced into the binary endpoint. Their predicted probabilities overlapped both Sensitive and Resistant groups rather than forming a clean intermediate band. Among the 61 binary matches, the historical affinity label and the later cellular classification agreed for 19 records (31.1%) after harmonizing the synonymous labels “Susceptible” and “Sensitive.” This low agreement and the poor calibration transfer are consistent with differences between an affinity-change benchmark and a later cellular prime-editing resistance assay. The two outcome systems should therefore not be treated as interchangeable ground-truth labels.

**Fig 8:**
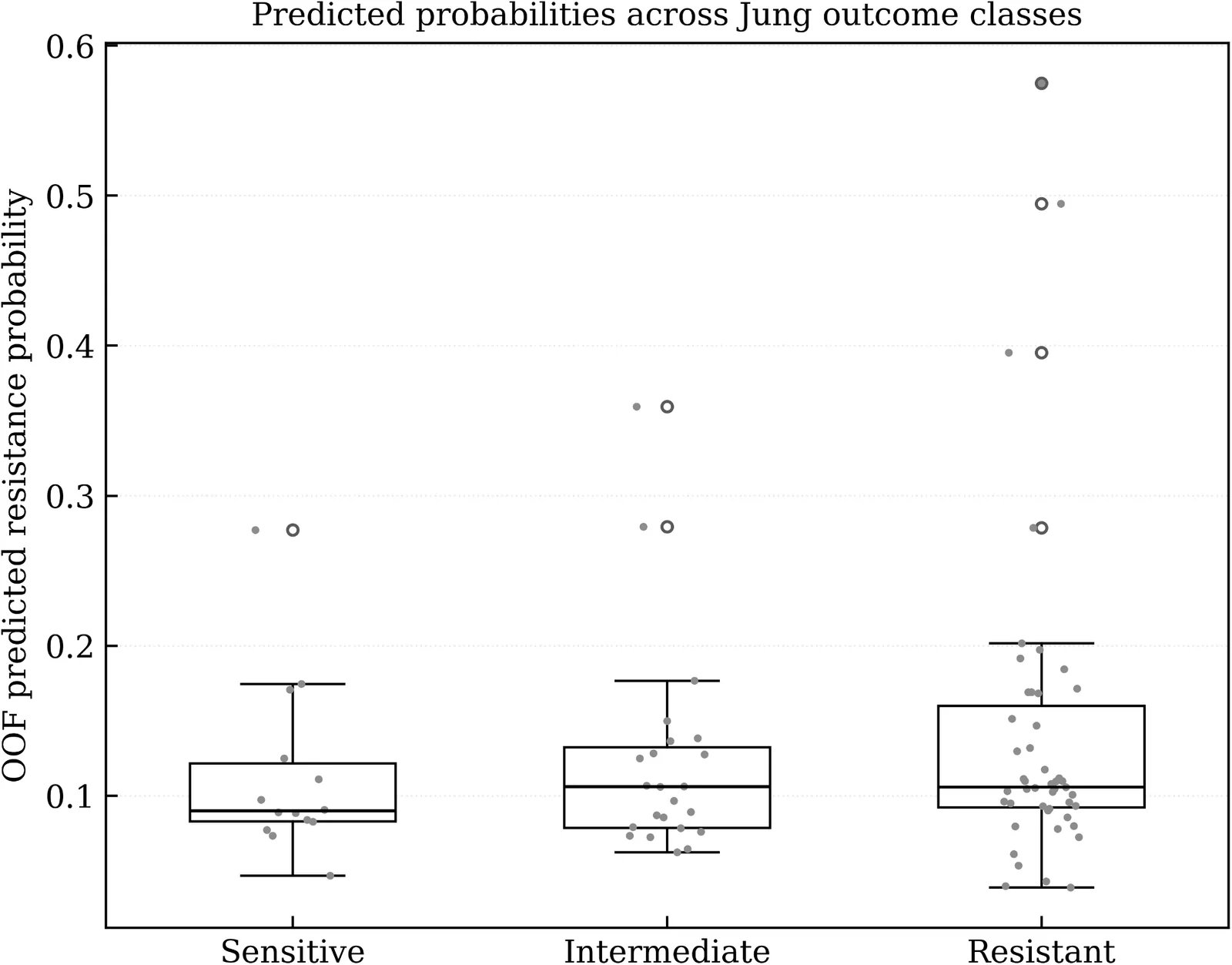
OOF resistance probabilities across Jung outcome classes. Sensitive, Intermediate, and Resistant classifications are shown separately. Intermediate outcomes are not used in the primary binary performance metrics.

Per-TKI temporal AUROCs for the stack ranged from 0.467 to 0.543 across the four overlapping inhibitors. These drug-specific estimates were based on only 12–19 records per TKI and are therefore reported descriptively in S4 Results rather than interpreted as stable inhibitor-level performance estimates.

## Discussion

Mutation-disjoint grouped OOF validation yielded an AUROC of 0.645 for the 11-feature stack, with substantial variation across repeated group assignments. The wide confidence interval and repeat-to-repeat variability indicate considerable uncertainty in generalization estimates from a 144-record dataset containing only 19 resistant examples. These results emphasize the importance of mutation-level separation when evaluating structure-aware resistance models.

The Jung dataset provides a temporally subsequent and experimentally distinct outcome source. The 84 mapped records represent exact mutation–drug identities rather than completely unseen feature vectors, so the 61-record binary analysis quantifies temporal cross-study outcome transport rather than unseen-variant input generalization. The 11-feature stack showed modest discrimination (AUROC 0.605) and poor probability calibration, while Elastic-Net achieved a numerically higher but statistically uncertain AUROC. These results indicate that greater model complexity does not necessarily improve transport under domain shift.

The discrepancy between the historical affinity-based endpoint and the Jung cellular classification is scientifically informative. The original benchmark was constructed from mutation-induced changes in inhibitor affinity using a ten-fold threshold [8], whereas Jung et al. quantified resistance in a high-throughput K562 prime-editing system [27]. These outcomes reflect different biological levels, assay conditions, and prevalence structures. A model calibrated to one endpoint is therefore not expected to preserve absolute probability calibration automatically in the other. The large calibration intercept and shallow slope observed here quantify that failure of transport directly.

These findings also clarify the relationship between machine learning and molecular biophysics. The structural predictors used here encode local geometry, protein–ligand contacts, Vina terms, solvent exposure, and Rosetta energy components derived from a public benchmark workflow [15]. Such descriptors can provide mechanistically interpretable summaries at lower computational cost than atomistic free-energy calculations, but they should not be described as replacements for FEP or molecular dynamics. Hauser et al.’s FEP benchmark remains a stronger physics-based reference on the historical data [8]. The present results instead support a complementary role: structure-aware machine learning may prioritize hypotheses, whereas FEP/MD can test specific energetic mechanisms in higher detail.

Feature-space interpolation and counterfactual perturbation of engineered descriptors are useful for sensitivity analysis but do not generate physically realized mutant structures. Accordingly, such synthetic perturbations are not used as confirmatory evidence for mutation mechanisms in this study. Evaluation of unseen variants would require generation of the same structural descriptors from explicit mutant complexes, documentation of conformational sampling and uncertainty, and prospective or temporally independent experimental evaluation.

Related public-bioactivity work using a larger harmonized BindingDB/ChEMBL dataset also showed lower performance under source-aware database transfer than under random splitting [26]. Together, these findings indicate that resistance prediction is sensitive to how records, mutations, assays, and data sources are partitioned. Validation design is therefore a major determinant of the apparent reliability of mutation–TKI screening models.

## Limitations

Several limitations remain. First, the historical benchmark is small and strongly imbalanced, with only 19 resistant records, producing wide mutation-cluster bootstrap intervals. Second, the fixed 11-feature panel was derived from the same historical benchmark in published work; internal OOF performance therefore reflects robustness of a predefined panel rather than a fully nested estimate of feature discovery. The secondary bootstrap analysis further showed that stability-selected subsets depend on Elastic-Net regularization settings. Third, the temporal validation contains only 21 overlapping mutation identities across four TKIs and is enriched for resistant outcomes, limiting calibration assessment and making PR-AUC strongly prevalence-dependent. Fourth, the Jung endpoint reflects cellular resistance rather than the affinity-change endpoint used in the historical benchmark, so temporal validation spans both time and assay definition. Fifth, structural descriptors were inherited from the public Yang et al. benchmark and were not recomputed in this study; uncertainty from structure preparation, protonation, docking, force-field settings, and conformational sampling is therefore not independently estimated. Sixth, the mapped Jung records correspond to mutation–drug identities already represented in the historical feature matrix and do not constitute unseen-variant validation. Finally, the predictions have not been prospectively evaluated for patient-level treatment selection.

## Conclusion

Mutation-disjoint grouped OOF evaluation produced moderate and unstable discrimination, and later prime-editing outcomes showed modest temporal transportability with substantial probability-calibration shift. Simpler regularized modelling was at least as competitive as the 11-feature stack under external shift. These findings support a screening use case in which structure-aware machine learning prioritizes resistance hypotheses and identifies patterns for experimental follow-up, while the predicted probabilities are not interpreted as clinically calibrated treatment recommendations. Future work should use prospectively defined mutation-disjoint datasets, generate structural descriptors explicitly for unseen variants, compare predictions directly with physics-based estimates on matched cases, and evaluate recalibration in independent validation cohorts.

## Supporting information

S1 Data Historical 144-record mutation-disjoint OOF predictions and model probabilities. (CSV)

S2 Data Exact mapping of 84 historical mutation–TKI identities to Jung later-study classifications and OOF predictions. (CSV)

S3 Data Primary 61-record sensitive/resistant temporal validation subset. (CSV)

S4 Results Internal and temporal performance, bootstrap confidence intervals, threshold analyses, per-TKI results, calibration bins, and repeat stability. (XLSX)

S5 Methods Fixed predictor-panel provenance and Elastic-Net selection-frequency sensitivity analysis. (XLSX)

S6 Code Python code recalculating reported metrics, calibration summaries, paired cluster-bootstrap contrasts, and the internal calibration figure from the archived record-level OOF predictions, together with a reference implementation of the stated grouped OOF training pipeline. (ZIP)

## References

[1] Druker BJ, Talpaz M, Resta DJ, Peng B, Buchdunger E, Ford JM, et al. Efficacy and safety of a specific inhibitor of the BCR-ABL tyrosine kinase in chronic myeloid leukemia. N Engl J Med. 2001;344(14):1031–7. doi: 10.1056/NEJM200104053441401 11287972

[2] O’Brien SG, Guilhot F, Larson RA, Gathmann I, Baccarani M, Cervantes F, et al. Imatinib compared with interferon and low-dose cytarabine for newly diagnosed chronic-phase chronic myeloid leukemia. N Engl J Med. 2003;348(11):994–1004. doi: 10.1056/NEJMoa022457 12637609

[3] O’Hare T, Shakespeare WC, Zhu X, Eide CA, Rivera VM, Wang F, et al. AP24534, a pan-BCR-ABL inhibitor for chronic myeloid leukemia, potently inhibits the T315I mutant and overcomes mutation-based resistance. Cancer Cell. 2009;16(5):401–12. doi: 10.1016/j.ccr.2009.09.028 19878872 PMC2804470

[4] Hughes TP, Mauro MJ, Cortes JE, Minami H, Rea D, DeAngelo DJ, et al. Asciminib in chronic myeloid leukemia after ABL kinase inhibitor failure. N Engl J Med. 2019;381(24):2315–26. doi: 10.1056/NEJMoa1902328 31826340 PMC7724923

[5] Soverini S, Hochhaus A, Nicolini FE, Gruber F, Lange T, Saglio G, et al. BCR-ABL kinase domain mutation analysis in chronic myeloid leukemia patients treated with tyrosine kinase inhibitors: Recommendations from an expert panel on behalf of European LeukemiaNet. Blood. 2011;118(5):1208–15. doi: 10.1182/blood-2010-12-326405 21562040

[6] Hochhaus A, Baccarani M, Silver RT, Schiffer C, Apperley JF, Cervantes F, et al. European LeukemiaNet 2020 recommendations for treating chronic myeloid leukemia. Leukemia. 2020;34(4):966–84. doi: 10.1038/s41375-020-0776-2 32127639 PMC7214240

[7] Braun TP, Eide CA, Druker BJ. Response and resistance to BCR-ABL1-targeted therapies. Cancer Cell. 2020;37(4):530–42. doi: 10.1016/j.ccell.2020.03.006 32289275 PMC7722523

[8] Hauser K, Negron C, Albanese SK, Ray S, Steinbrecher T, Abel R, et al. Predicting resistance of clinical Abl mutations to targeted kinase inhibitors using alchemical free-energy calculations. Commun Biol. 2018;1:70. doi: 10.1038/s42003-018-0075-x 30159405 PMC6110136

[9] Zhou Y, Portelli S, Pat M, Rodrigues CHM, Nguyen T-B, Pires DEV, et al. Structure-guided machine learning prediction of drug resistance mutations in Abelson 1 kinase. Comput Struct Biotechnol J. 2021;19:5381–91. doi: 10.1016/j.csbj.2021.09.016 34667533 PMC8495037

[10] Trott O, Olson AJ. AutoDock Vina: improving the speed and accuracy of docking with a new scoring function, efficient optimization, and multithreading. J Comput Chem. 2010;31(2):455–61. doi: 10.1002/jcc.21334 19499576 PMC3041641

[11] Schymkowitz J, Borg J, Stricher F, Nys R, Rousseau F, Serrano L. The FoldX web server: An online force field. Nucleic Acids Res. 2005;33(Web Server issue):W382-8. doi: 10.1093/nar/gki387 15980494 PMC1160148

[12] Alford RF, Leaver-Fay A, Jeliazkov JR, O’Meara MJ, DiMaio FP, Park H, et al. The Rosetta all-atom energy function for macromolecular modeling and design. J Chem Theory Comput. 2017;13(6):3031–48. doi: 10.1021/acs.jctc.7b00125 28430426 PMC5717763

[13] Dhanasekaran S, Selvadoss PP, Manoharan SS, Jeyabalan S, Muthu Laxmi V, Choudhury AA, et al. Targeting nudix hydrolase 5 with bioactive flavonoids: Molecular dynamics and docking studies for breast cancer therapy. Cell Biochem Biophys. 2025;83(2):1973–91. doi: 10.1007/s12013-024-01609-x 39638981

[14] Pires DEV, Blundell TL, Ascher DB. mCSM-lig: quantifying the effects of mutations on protein-small molecule affinity in genetic disease and emergence of drug resistance. Sci Rep. 2016;6:29575. doi: 10.1038/srep29575 27384129 PMC4935856

[15] Yang Z-Y, Ye Z-F, Xiao Y-J, Hsieh C-Y, Zhang S-Y. SPLDExtraTrees: Robust machine learning approach for predicting kinase inhibitor resistance. Brief Bioinform. 2022;23(3):bbac050. doi: 10.1093/bib/bbac050 35262669

[16] Mayr A, Klambauer G, Unterthiner T, Steijaert M, Wegner JK, Ceulemans H, et al. Large-scale comparison of machine learning methods for drug target prediction on ChEMBL. Chem Sci. 2018;9(24):5441–51. doi: 10.1039/c8sc00148k 30155234 PMC6011237

[17] Vamathevan J, Clark D, Czodrowski P, Dunham I, Ferran E, Lee G, et al. Applications of machine learning in drug discovery and development. Nat Rev Drug Discov. 2019;18(6):463–77. doi: 10.1038/s41573-019-0024-5 30976107 PMC6552674

[18] Moshawih S, Bu ZH, Goh HP, Kifli N, Lee LH, Goh KW, et al. Consensus holistic virtual screening for drug discovery: A novel machine learning model approach. J Cheminform. 2024;16(1):62. doi: 10.1186/s13321-024-00855-8 38807196 PMC11134635

[19] Wallach I, Heifets A. Most ligand-based classification benchmarks reward memorization rather than generalization. J Chem Inf Model. 2018;58(5):916–32. doi: 10.1021/acs.jcim.7b00403 29698607

[20] Varma S, Simon R. Bias in error estimation when using cross-validation for model selection. BMC Bioinformatics. 2006;7:91. doi: 10.1186/1471-2105-7-91 16504092 PMC1397873

[21] Sheridan RP. Time-split cross-validation as a method for estimating the goodness of prospective prediction. J Chem Inf Model. 2013;53(4):783–90. doi: 10.1021/ci400084k 23521722

[22] Varoquaux G. Cross-validation failure: Small sample sizes lead to large error bars. Neuroimage. 2018;180(Pt A):68–77. doi: 10.1016/j.neuroimage.2017.06.061 28655633

[23] Saito T, Rehmsmeier M. The precision-recall plot is more informative than the ROC plot when evaluating binary classifiers on imbalanced datasets. PLoS One. 2015;10(3):e0118432. doi: 10.1371/journal.pone.0118432 25738806 PMC4349800

[24] Van Calster B, McLernon DJ, van Smeden M, Wynants L, Steyerberg EW, Topic Group ‘Evaluating diagnostic tests and prediction models’ of the STRATOS initiative. Calibration: the Achilles heel of predictive analytics. BMC Med. 2019;17(1):230. doi: 10.1186/s12916-019-1466-7 31842878 PMC6912996

[25] Hassan F, Deif MA, Zaghloul A, Elgohary R, Khishe M. Explainable stacked-ensemble prediction of ABL1 tyrosine-kinase inhibitor resistance: A metaheuristic-optimized pipeline. Intell-Based Med. 2026;13:100341. doi: 10.1016/j.ibmed.2025.100341

[26] Deif MA, Hafez MA, Khishe M. Calibrated stacked learning for source-aware screening of ABL1/BCR–ABL1 mutation–TKI resistance. Egypt Inform J. 2026;35:101045. doi: 10.1016/j.eij.2026.101045

[27] Jung Y, Yu G, Oh H-C, Lee J-H, Jeon E, Bae J, et al. Comprehensive resistance profiling of chronic myeloid leukaemia associated ABL1 variants against five tyrosine kinase inhibitors using prime editing. Nat Biomed Eng. 2026;10(7):1392–409. doi: 10.1038/s41551-025-01531-4 41125871

[28] Salentin S, Schreiber S, Haupt VJ, Adasme MF, Schroeder M. PLIP: Fully automated protein-ligand interaction profiler. Nucleic Acids Res. 2015;43(W1):W443–7. doi: 10.1093/nar/gkv315 25873628 PMC4489249

